# Al_2_O_3_-based nanofluids: a review

**DOI:** 10.1186/1556-276X-6-456

**Published:** 2011-07-16

**Authors:** Veeranna Sridhara, Lakshmi Narayan Satapathy

**Affiliations:** 1 New Horizon College of Engineering, Bangalore, India; 2 Ceramic Technological Institute, BHEL, Malleswaram Complex, Bangalore 560012, India

## Abstract

Ultrahigh performance cooling is one of the important needs of many industries. However, low thermal conductivity is a primary limitation in developing energy-efficient heat transfer fluids that are required for cooling purposes. Nanofluids are engineered by suspending nanoparticles with average sizes below 100 nm in heat transfer fluids such as water, oil, diesel, ethylene glycol, etc. Innovative heat transfer fluids are produced by suspending metallic or nonmetallic nanometer-sized solid particles. Experiments have shown that nanofluids have substantial higher thermal conductivities compared to the base fluids. These suspended nanoparticles can change the transport and thermal properties of the base fluid. As can be seen from the literature, extensive research has been carried out in alumina-water and CuO-water systems besides few reports in Cu-water-, TiO_2_-, zirconia-, diamond-, SiC-, Fe_3_O_4_-, Ag-, Au-, and CNT-based systems. The aim of this review is to summarize recent developments in research on the stability of nanofluids, enhancement of thermal conductivities, viscosity, and heat transfer characteristics of alumina (Al_2_O_3_)-based nanofluids. The Al_2_O_3 _nanoparticles varied in the range of 13 to 302 nm to prepare nanofluids, and the observed enhancement in the thermal conductivity is 2% to 36%.

## Introduction

Conventional fluids, such as water, engine oil, and ethylene glycol are normally used as heat transfer fluids. Although various techniques are applied to enhance the heat transfer, the low heat transfer performance of these conventional fluids obstructs the performance enhancement and the compactness of heat exchangers. The use of solid particles as an additive suspended into the base fluid is technique for the heat transfer enhancement. Improving the thermal conductivity is the key idea to improve the heat transfer characteristics of conventional fluids. Since a solid metal has a larger thermal conductivity than a base fluid, suspending metallic solid fine particles into the base fluid is expected to improve the thermal conductivity of that fluid. The enhancement of thermal conductivity of conventional fluids by the suspension of solid particles, such as millimeter- or micrometer-sized particles, has been well-known for many years [1]. However, they have not been of interest for practical applications due to problems such as sedimentation leading to increased pressure drop in the flow channel. The recent advance in material technology has made it possible to produce innovative heat transfer fluids by suspending nanometer-sized particles in base fluids which can change the transport and thermal properties of the base fluid.

Nanofluids are solid-liquid composite materials consisting of solid nanoparticles or nanofibers with sizes typically of 1 to 100 nm suspended in liquid. The nanofluid is not a simple liquid-solid mixture; the most important criterion of nanofluid is agglomerate-free stable suspension for long durations without causing any chemical changes in the base fluid. This can be achieved by minimizing the density between solids and liquids or by increasing the viscosity of the liquid; by using nanometer-sized particles and by preventing particles from agglomeration, the settling of particles can be avoided. Nanofluids have attracted great interest recently because of reports of enhanced thermal properties [2-6]. Extensive research has been carried out on alumina-water- and CuO-water-based systems besides few reports in Cu-water, carbon nanotubes water systems.

This article aims at an overview of the concept of "alumina-based Nanofluids" followed by an account on the detailed research activities carried out around the world. The review will focus mainly on engineering application parameters, such as thermal conductivity and viscosity etc., without giving much emphasis on the theoretical aspects.

### Preparation of nanofluids

There are two fundamental methods to obtain nanofluids:

1. Single-step direct evaporation method: In this method, the direct evaporation and condensation of the nanoparticulate materials in the base liquid are obtained to produce stable nanofluids.

2. Two-step method: In this method, first the nanoparticles are obtained by different methods and then are dispersed into the base liquid.

### Thermal conductivity measurement techniques

Thermal conductivity is an important parameter in enhancing the heat transfer performance of a base fluid. Since the thermal conductivity of solid metals is higher than that of fluids, the suspended particles are expected to increase the thermal conductivity and heat transfer performance. Many researchers have reported experimental studies on the thermal conductivity of nanofluids. The temperature oscillation method [7], the steady-state parallel plate method [8], and transient hot-wire method [1] have been employed to measure the thermal conductivity of nanofluids. However, the transient hot-wire method has been extensively used by many researchers [9-12]. A detailed review on different techniques for measurement of thermal conductivity of nanofluids is available in the literature [5].

### Experimental results on thermal conductivity of Al_2_O_3_-based nanofluids

Alumina (Al_2_O_3_) is the most common nanoparticle used by many researchers in their experimental works. Many efforts have been made to study the thermal conductivity of nanofluids. The summary of experimental studies on the thermal conductivity of Al_2_O_3_-based nanofluids are given in Table 1. Generally, thermal conductivity of the nanofluids increases with increasing volume fraction of nanoparticles; with decreasing particle size, the shape of particles can also influence the thermal conductivity of nanofluids, temperature, Brownian motion of the particle, interfacial layer, and with the additives.

**Table 1:** The selective summary of the thermal conductivity enhancement in Al_2_O_3_-based nanofluids

Author (year)	Base fluid	Concentration	Particle size (nm)	Enhancement ratio	Method/parameters
Masuda *et al*. [15]	Water (31.85°C) Water (46.85°C) Water (66.85°C)	1.3 to 4.3	13	1.1092 to 1.324 1.10 to 1.296 1.092 to 1.262	Two-step method Temperature effect
Lee *et al*. [1]	Water Ethylene	1.0 to 4.30 1.0 to 5.0	38.4	1.03 to 1.10 1.03 to 1.18	Two-step method
Wang *et al*. [8]	Water Ethylene glycol Engine oil Pump oil	3.0 to 5.50 5.0 to 8.0 2.25 to 7.40 5.00 to 7.10	28	1.11 to 1.16 1.25 to 1.41 1.05 to 1.30 1.13 to 1.20	Two-step method
Eastman *et al*. [18]	Ethylene glycol	1.00 to 5.00	35		Two-step method
Xie *et al*. [16]	Water Ethylene glycol Ethylene glycol Ethylene glycol Ethylene glycol Pump oil	1.80 to 5.00 1.80 to 5.00 1.80 to 5.00 1.80 to 5.00 1.80 to 5.00 5.00	60.4 15 26 60.4 302 60.4	1.07 to 1.21 1.06 to 1.17 1.06 to 1.18 1.10 to 1.30 1.08 to 1.25 1.39	Two-step method Solid crystalline Phase effect Morphology effect pH effect
Xie *et al*. [16]	Water Ethylene glycol Pump oil Glycerol	5.0 5.0 5.0 5.0	60.4	1.39 1.23 1.29 1.38	Two-step method Base fluid effect
Das *et al*. [7]	Water (21°C) Water (36°C) Water (51°C)	1.00 to 4.00	38.4	1.02 to 1.09	Two-step method Temperature effect
Wen and Ding [24]	Water + sodium dodecyl benezene sulfonate	0.19 to 1.59	42	1.01 to 1.10	Two-step method
Li and Peterson [13]	Water (27.5°C) Water (32.5°C) Water (34.7°C)	2.00 to 10.00	36	1.08 to 1.11 1.15 to 1.22 1.18 to 1.29	Two-step method Temperature effect
Beck *et al*. [11]	EG (27°C)	1.00 to 4.00	20	1.015 to 1.14	Two-step method
Hwang *et al*. [17]	Water	0.3 to 1.0	48	1.013 to 1.04	Two-step method
Timofeeva *et al*. [14]	Water EG	5.0 5.0 5.0 5.0	11 20 40 All sizes	1.08 1.07 1.10 1.13	Two-step method Temperature effect (296°C to 333°C)
Lee *et al*. [19]	Water	0.01 to 03	35	1.005 to 1.02	Two step
Murshed *et al*. [20]	Water EG CTAB	1.0 0.5 1.0	80 150 80 80	1.03 to 1.12 1.02 to 1.10 1.03 to 1.09 1.06 to 1.12	Two-step method Temperature range (21°C to 60°C)
Choi *et al*. [12]	Transformer oil + oleic acid	0.5 to 4.0	13	1.05 to 1.20	Two-step method
Oh *et al*. [23]	Water EG	1.0 to 4.0 1.0 to 4.0	45 45	1.044 to 1.133 1.019 to 1.097	Two-step method
Kole *et al*. [25]	Car engine coolant	3.5	50	1.1041 to 1.1125	Two-step method Temperature effect (30°C to 80°C)

### Effect of volume fraction of nanoparticles on thermal conductivity of Al_2_O_3_-based nanofluids

**Figure 1:**
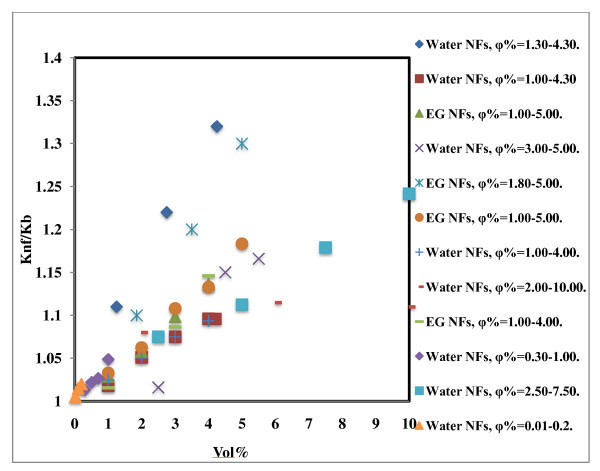
**Effect of concentration on thermal conductivity of Al_2_O_3_-based nanofluids**. Water NFs, φ% = 1.30-4.30 [15]; water NFs, φ% = 1.00-4.30 [1]; EG NFs, φ% = 1.00-5.00 [1]; water NFs, φ% = 3.00-5.00 [8]; EG NFs, φ% = 1.80-5.00 [16]; EG NFs, φ% = 1.00-5.00 [18]; water NFs, φ% = 1.00-4.00 [7]; water NFs, φ% = 2.00-10.00 [13]; EG NFs, φ% = 1.00-4.00 [11]; water NFs, φ% = 0.30-1.00 [17]; water NFs, φ% = 2.50-7.50 [14]; water NFs, φ% = 0.01-0.20 [19].

The effect of volume concentration on Al_2_O_3_-based nanofluids is shown in Figure 1. The researchers used different sizes of Al_2_O_3 _nanoparticles at different temperatures in water and ethylene glycol with particle volume concentration mostly less than 5% with few exceptions [13,14]. The maximum enhancement in thermal conductivity observed for 4 vol.% load in the case of water-based nanofluid was 32% [15] and in the case of ethylene glycol-based nanofluid was 30% [16], respectively. Hwang *et al*. [17] observed a 4% enhancement in thermal conductivity at 1 vol.% concentration; the observed enhancement was more compared to other researchers at same the volume fraction of solids [1,11,18]. Lee *et al*. [19] observed a 2% enhancement at a lower volume percent for 35-nm-sized Al_2_O_3 _particles. In the case of Li and Peterson [13], the thermal conductivity enhancement was decreased as concentration increased from 6% to 10%, but in the case of Timofeeva *et al*. [14], the thermal conductivity was increased as concentration increased from 2% to 10% even though the particle size was almost the same in both the cases.

### Effect of particle size on thermal conductivity of Al_2_O_3 _based nanofluids

**Figure 2:**
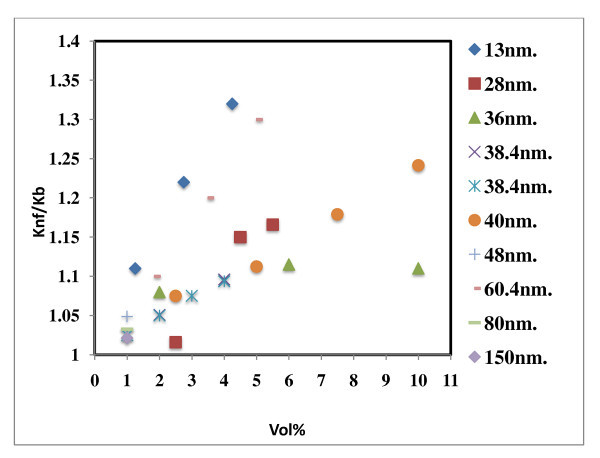
**Effect of particle size on thermal conductivity of water-based Al_2_O_3 _nanofluids**. 13 nm [15], 28 nm [8], 36 nm [13], 38.4 nm [1], 38.4 nm [7], 40 nm [14], 48 nm [17], 60.4 nm [16], 80 nm [20], 150 nm [20].

**Figure 3:**
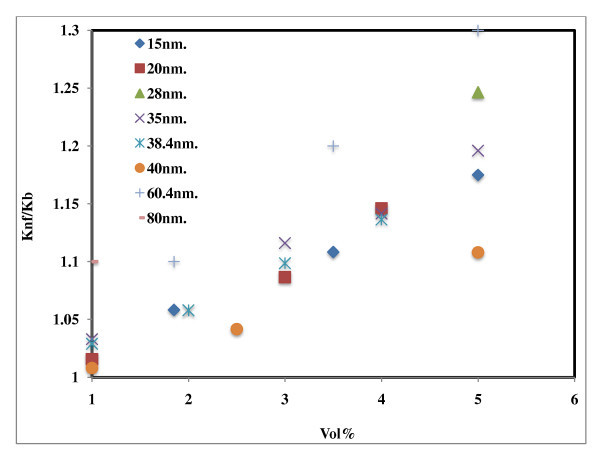
**Effect of particle size on thermal conductivity of ethylene glycol-based Al_2_O_3 _nanofluids**. 15 nm [16], 20 nm [11], 28 nm [8], 35 nm [18], 38.4 nm [1], 40 nm [14], 60.4 nm [16], 80 nm [20].

Figure 2 demonstrates the effect of particle size on thermal conductivity of Al_2_O_3_-based nanofluids; the particles used were in the range of 13 to 150 nm. Alumina, 38.4 nm, in water resulted in thermal conductivity enhancement in the range of 2% to 10% in two studies [1,7] but up to 21% in another study [16]. The thermal conductivity enhancement for the nanofluids with 28 nm [8] particles was lying in between that of 38.4 and 60.4 nm, which cannot be explained. Murshed *et al*. [20] observed higher enhancement with 80- and 150-nm-sized particles at 1 vol.% compared to the nanofluids with 2.5 vol.% of 28-nm particles in ethylene glycol-based Al_2_O_3 _nanofluids as shown in Figure 3. The authors have demonstrated that 80-nm particles showed higher thermal conductivity enhancement at 1 vol.% compared to similar data reported earlier [1,11,14]. Xie *et al*. [16] used 15- and 60.4-nm-sized particles, observed higher thermal conductivity enhancement for larger nanoparticles in ethylene glycol-based nanofluids. The results cited here do not correlate the size effect of nanoparticles in thermal conductivity enhancement. More research is required to understand this size effect.

### Effect of base fluids on thermal conductivity of Al_2_O_3_-based nanofluids

**Figure 4:**
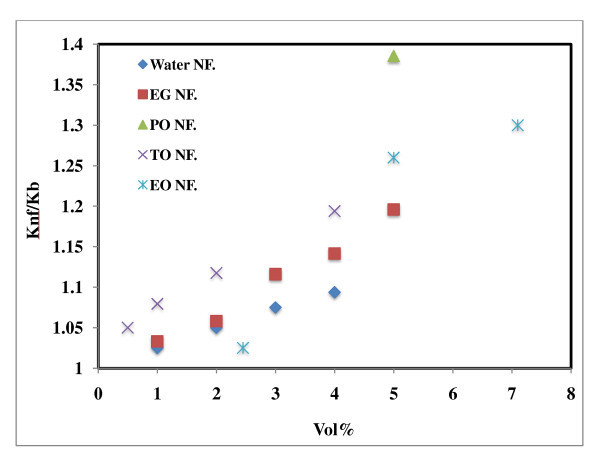
**Effect of base fluids on thermal conductivity of Al_2_O_3_-based nanofluids**. Water NF [7], EG NF [18], PO NF [16], TO NF [12], EO NF [8].

The effect of base fluid on thermal conductivity is shown in Figure 4. The result in Figure 4 demonstrates that the thermal conductivity enhancement is least for the water-based nanofluids compared with other nanofluids. This result is encouraging because heat transfer enhancement is often most needed when poorer heat transfer fluids are involved. The enhancement in the case of PO is 38% at 5 vol.% compared to that of 20% at 4 vol.% TO in contrast to 10.8% enhancement with the same volume fraction of nanoparticles in water [21]. Figure 4 thus categorically indicated that the thermal conductivity enhancement for the poorer heat transfer fluids is good compared to the fluids with better thermal conductivity such as water.

### Effect of preparation method on thermal conductivity of Al_2_O_3_-based nanofluids

Thermal conductivities of the nanoparticle fluid mixture were first reported by Masuda *et al*. [15]. The mean diameter of the particles used in their experiments was 13 nm, and the particles dispersed in water by using a high-speed shearing dispenser ≈ 20,000 rpm. The authors reported a 32.4% increase in thermal conductivity for the volume fraction of 4.3 vol.% against 20% for 3 vol.% nano alumina. However, the experiment was carried out at a higher room temperature of approximately 32°C, which is higher than most other researchers' reported data at room temperature ranging from 21°C to 28°C. Further, the authors used a high-speed dispenser with addition of HCl and NaOH to the fluids so that electrostatic repulsive forces among the particles kept the powder well dispersed. Lee *et al*. [1] dispersed 38.4-nm-sized Al_2_O_3 _nanoparticles in water and ethylene glycol by using polyethylene container and shaken thoroughly to ensure a homogeneous suspension for producing stable suspension. The authors observed an increase of only 10% at the 4.3 vol.% and 8% for the 3% load. The same enhancement was observed by Das *et al*. [7] for the particle size of 38.4 nm and for the particle load between 1% and 4%. Wang *et al*. [8] dispersed 28-nm-sized Al_2_O_3 _nanoparticles in different base fluids and prepared nanofluids by mechanical blending, coating particles with polymers and filtration method. The thermal conductivity enhancement was 16% for 5.5 vol.% and 12% for 3% volume faction. In the case of Xie *et al*. [16], the researchers used 60.4-nm-sized Al_2_O_3 _dispersed in water and prepared stable solution by adjusting pH. The nanoparticles are de-agglomerated by using an ultrasonic disrupter after mixing with a base fluid and were homogenized by using magnetic force agitation. The enhancement observed was 21% for 5% volume fraction and 14% at 3.2% volume fraction.

**Figure 5:**
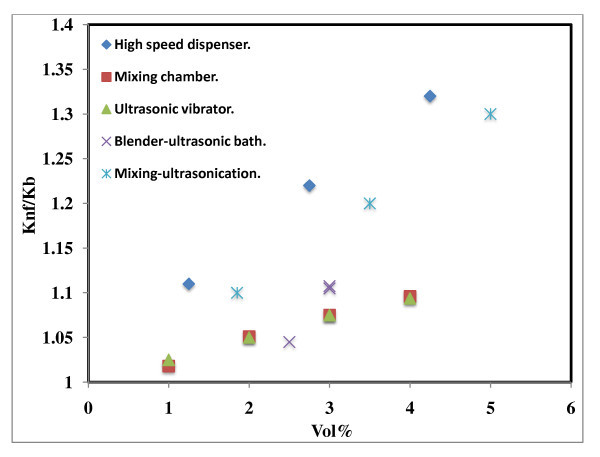
**Effect of preparation techniques on thermal conductivity of Al_2_O_3_-based nanofluids**. High-speed disperser [15], mixing chamber [1], ultrasonic vibrator [7], blender-ultrasonic bath [8], mixing-ultrasonication [16].

Figure 5 shows that the enhancement in the case of Xie *et al*. [16] is more compared to others even though they used lesser particles and in the case of Wang *et al*. [8] shows lesser enhancement at 2.5 vol.% compared to Das *et al*. [7] and Lee *et al*. [1]. The same method of synthesis in the latter two cases demonstrated similar enhancement ratios. These results demonstrate that a stable dispersion can be achieved by many different methods, but the thermal conductivity enhancement is dependent on the preparation methods. These results need further studies since the stability of such fluids in the long run has not been studied and the data reported here is immediately after obtaining the nanofluid.

### Effect of temperature on thermal conductivity of Al_2_O_3_-based nanofluids

**Figure 6:**
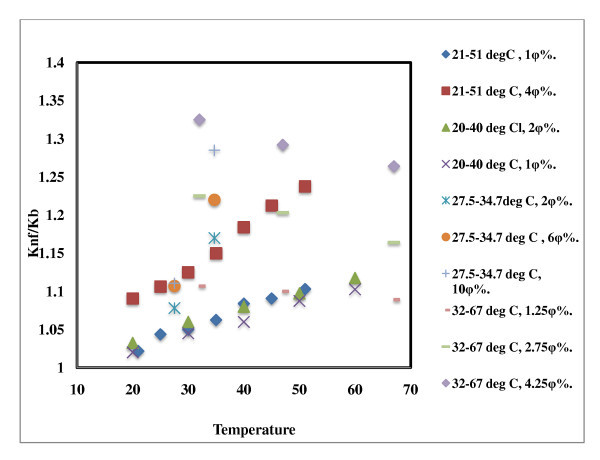
**Effect of temperature on thermal conductivity of Al_2_O_3_-based nanofluids**. 21°C to 51°C, 1 φ% [7]; 21°C to 51°C, 4 φ% [7]; 20°C to 40°C, 2 φ% [20]; 20°C to 40°C, 1 φ% [20]; 27.5°C to 34.7°C, 2 φ% [13]; 27.5°C to 34.7°C, 6 φ% [13]; 27.5°C to 34.7°C, 10 φ% [13]; 52°C to 67°C, 1.25 φ% [15]; 52°C to 67°C, 2.75 φ% [15]; 52°C to 67°C, 4.25 φ% [15].

The thermal conductivity of nanofluids is temperature sensitive compared to that of base fluids. The effect of temperature on water-based Al_2_O_3 _nanofluids is shown in Figure 6. Different groups measured thermal conductivity at different temperatures. Das *et al*. [7] varied temperatures in the range of 21°C to 51°C demonstrating an enhancement of 2% to 10.8% for the particle load of 2 vol.% and observed thermal conductivity enhancement of 9.4% as compared to 24.3% for 4 vol.% solids. The authors suggested that strong temperature dependence of nanofluid thermal conductivity is due to the motion of the particles. The larger sized particles used by Murshed *et al*. [20] resulted in enhancement similar to that reported earlier [7] indicating that the enhancement is due to the intensification of the Brownian motion of the nanoparticles by addition of a surfactant and the application of temperature. The general trend in Figure 6 of increased thermal conductivity enhancement with increased temperature is not in line with a very early report of Masuda *et al*. [15].

**Figure 7:**
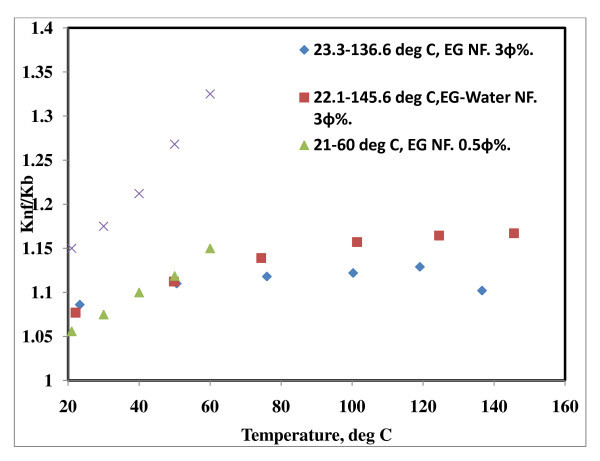
**Effect of temperature on EG and EG + H_2_O-based Al_2_O_3 _nanofluids**. 23.3 to 136.6, EG NF, 3 φ% [22]; 222.1 to 145.6, EG-water NF, 3 φ% [22]; 21°C to 60°C, EG NF, 0.5 φ% [20]; 21°C to 60°C, EG NF, 1.0 φ% [20].

In Figure 7, the same trend is observed for ethylene glycol-based nanofluids. Both Murshed *et al*. [20] and Beck *et al*. [22] observed higher conductivity enhancement for the suspensions containing surfactants, though particle size of solids were different in both cases. Recently, Beck *et al*. [22] measured thermal conductivity of the ethylene glycol-based nanofluids in the range of 296 to 400 K and showed that thermal conductivity behavior of nanofluids is related the behavior of the base fluid, and they suggested that temperature dependence of nanofluids is due mostly to the base fluids.

These results of temperature dependence of thermal conductivity enhancement in nanofluids based on alumina during the last 15 years since 1993 is confusing and hence needs thorough analysis. The data must be interpreted in conjunction with the base fluid behavior, particle size, and surfactant effect.

### Thermal conductivity of Al_2_O_3 _nanofluids measured by different techniques

**Figure 8:**
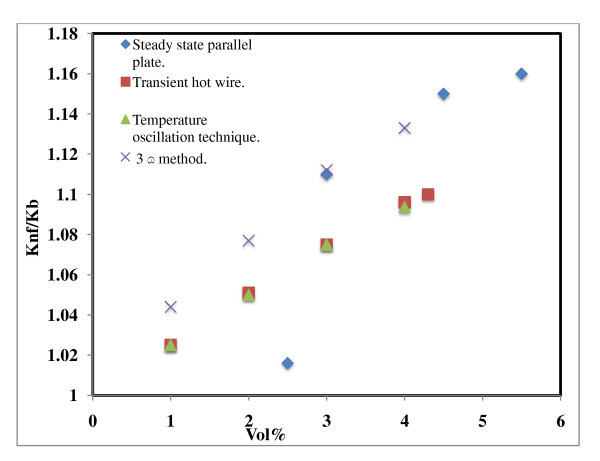
**Thermal conductivity of Al_2_O_3 _nanofluids measured by different techniques**. Steady-state parallel plate [8]; transient hot-wire method [1]; temperature oscillation technique [7]; 3ω method [23].

Figure 8 shows the thermal conductivity measurement of Al_2_O_3 _water-based nanofluid measured by different techniques. A trend shows that thermal conductivity increased with the increase in volume fraction. The thermal conductivity data in the case of Oh *et al*. [23] were in well agreement with that reported by Wang *et al*. [8] which, however, was higher than the results of Lee *et al*. [1] and Das *et al*. [7] for similar nanofluids but measured by different techniques. The reason for this discrepancy during the measurement may be due to the sedimentation and aggregation of nanoparticles, particle diameter, and nanofluid preparation. In comparing the thermal conductivity measurement techniques, the steady state parallel plate method seems to be least affected by the particle sedimentation since the thickness of the loaded sample fluid is less than 1 mm. The transient hot-wire method can be affected by the sedimentation of the nanofluids. Non-homogeneous nanoparticle concentration in the direction of gravity can give rise to temperature gradient within the vertical hot wire, which may be a source of measurement errors. This is also true for the temperature oscillation technique [23]. It is not clear how these techniques will behave for a stable nanofluid which does not at all sediment during the measurement. Therefore, it is essential to produce nanofluids which can be stable for long periods of time without any noticeable sedimentation.

### Effect pH on thermal conductivity of Al_2_O_3 _water-based nanofluids

**Figure 9:**
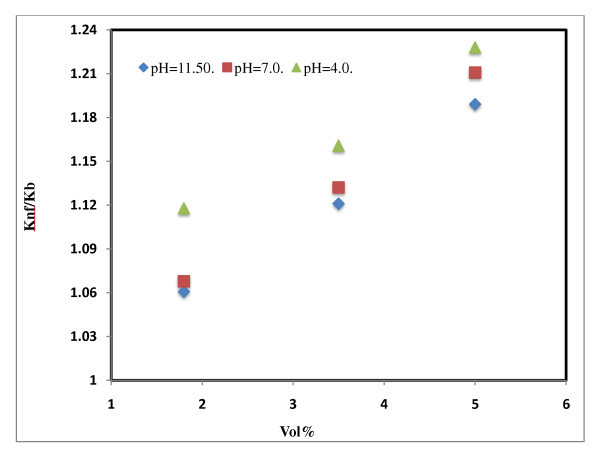
**Effect of pH on thermal conductivity of water-based Al_2_O_3 _nanofluids**. pH = 11.5 [16]; pH = 7.0 [16]; pH = 4.0 [16].

Xie *et al*. [16] prepared various suspensions containing Al_2_O_3 _nanoparticles with specific surface areas in a range of 5 to 124 m^2^/g, and their thermal conductivities were measured using a transient hot-wire method at a pH range of 2 to 11.5. It was noted that the nanoparticle suspensions, containing a small amount of Al_2_O_3_, have substantially higher thermal conductivity than the base fluid, with the enhancement increasing with the volume fraction of Al_2_O_3_. The enhanced thermal conductivity increases with an increase in the difference between the pH value of aqueous suspension and the isoelectric point of the Al_2_O_3 _particle. The enhancement observed for 60.4-nm-sized particle between 1.8 and 5 vol.% is 7% to 21%. The effect of pH on thermal conductivity of water-based Al_2_O_3 _nanofluids is shown in Figure 9.

### Effect of surface active agents on thermal conductivity of water-based Al_2_O_3 _nanofluids

**Figure 10:**
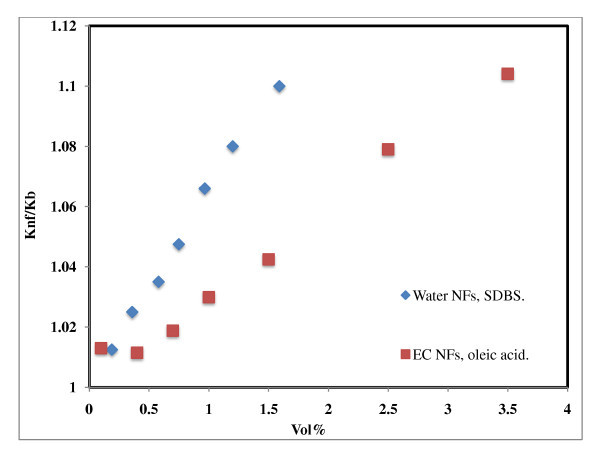
**Effect of additives on thermal conductivity of Al_2_O_3 _nanofluids**. Water NFs, SDBS [24]; EC NFs, oleic acid [25].

Figure 10 compares the thermal conductivity enhancement of Al_2_O_3 _nanofluids with and without a surfactant. Wen *et al*. [24] used 42-nm-sized Al_2_O_3 _nanoparticles and dispersed them in water using sodium dodecyl benzene sulfonate (SDBS) as surfactant; the enhancement observed was 10% for 1.59 vol.% which is comparable with the data reported earlier [1,7,18]. Recently, Kole *et al*. [25] dispersed < 50-nm-sized Al_2_O_3 _using oleic acid as surfactant in a car engine coolant and observed 10.41% enhancement for 3.5 vol.%. The authors have demonstrated the stability of such fluids for more than 80 days with thermal conductivity enhancement of 13% and 12% for ethylene glycol-based Al_2_O_3 _nanofluids at 5 vol.% solid loading. As shown in Figure 10, the additives will enhance the thermal conductivity of the nanofluids and give good stability, but the question which is unresolved is the contribution of thermal conductivity enhancement from the surfactant effect to the overall enhancement of the nanofluids.

### Experimental results on viscosity of Al_2_O_3_-based nanofluids

Compared with the experimental studies on thermal conductivity of nanofluids, there are limited rheological studies reported in the literature. In one study [8], the Al_2_O_3_-water mixture showed a viscosity increase between 20% and 30% for 3 vol.% Al_2_O_3 _solution compared to that of water alone. The results by Das *et al*. [26] on the viscosity of alumina-water nanofluids against shear rate demonstrated an increase of viscosity with increased particle concentrations indicating strong possibility that nanofluid may be non-Newtonian. Further investigations are, however, required to define the viscosity models of nanofluids.

In another study, a two-step method was used to produce Al_2_O_3_-water nanofluids with low concentrations of Al_2_O_3 _nanoparticles from 0.01 to 0.3 vol.% without any surfactant [19] and measured viscosity at the temperature range from 21°C to 39°C. Experimental results showed that the effective viscosities of the dilute Al_2_O_3_-water nanofluids significantly decreases with increasing temperature and slightly increases with increasing volume fraction. The measured viscosity of the Al_2_O_3_-water nanofluids is nonlinear with the Al_2_O_3 _nanoparticle volume concentration. The nonlinear viscosity behavior occurs at very low particle concentrations far below 2 vol.%. Nonlinear behavior implies that there is particle-particle interactions which invalidate the Einstein equation developed for dilute suspensions. The result is similar in another experiment [27], wherein, the viscosity increased by 83.4% at a volume fraction of 0.05 (5 vol.%). The viscosity study on Al_2_O_3_-water nanofluids with 36- and 47-nm, and CuO-water nanofluid with 29-nm average particle size was reported by Nguyen *et al*. [28] for particle volume fraction ranging from 1% to 9.4% and for temperatures varying from room temperature to approximately 75°C. The hysteresis behavior for 36-nm particle size and four particle volume concentrations indicated drastic changes with heating of samples beyond a critical temperature. On cooling after being heated beyond a critical temperature, a hysteresis phenomenon can occur. It is very interesting to note that hysteresis is predominant only in fluids with higher nanoparticle concentration.

The measured viscosities of Al_2_O_3 _(80 nm) and deionized water (DIW)-based nanofluids were also found to increase by nearly 82% for the maximum volumetric loading of 5% nanoparticles [20]. A similar increment (86%) of the effective viscosity of Al_2_O_3 _(28 nm)/distilled water-based nanofluids was also observed by Wang *et al*. [8] for the same volume fraction of 0.05. The reasons for the differences could be due to the difference in the size of the particle clusters, differences in the dispersion techniques, and the use of a surfactant similar to that reported earlier for thermal conductivity data. At lower concentrations, the change in relative viscosity over temperature was minimal. Xie *et al*. [29] also demonstrated that the viscosity of the nanoparticle suspension is much larger than the corresponding value predicted by the theoretical formula. The enhancements ratio of the viscosity of ethylene glycol (EG)-based suspensions are smaller than those of water-based suspensions, indicating the significant influence of the base fluid on the viscosity of the fluid-nanoparticle mixtures. The recent report by Kole *et al*. [25] of alumina in engine oil demonstrated that there is a transition from Newtonian characteristics for the base fluid to non-Newtonian behavior with increasing content of Al_2_O_3 _in the engine coolant. The data also show that the viscosity increases with an increase in concentration and decreases with an increase in temperature.

The analysis of limited data indicated that an optimization is required for the solid loading in nanofluids so that the viscosity rise is not high for the application and at the same time there is significant enhancement in the thermal conductivity of nanofluids. More studies are required in this direction.

### Heat transfer characteristics of Al_2_O_3_-based nanofluids

While heat transfer aspects of suspensions are important in applications in general, the aspect of natural convection in multiphase emulsions becomes more critical during storage and special phenomena such as melting of clathrate, which is used for storing coldness by releasing latent heat, separates out as organic liquid and an emulsion of hydrofluorocarbon dispersed in water [4].

Pak and Cho [30] studied the heat transfer enhancement in a circular tube, using γ-Al_2_O_3 _and TiO_2 _nanoparticle fluid mixtures as the flowing medium. They observed an increase in the Nusselt number with the increasing volume fraction and Reynolds number. Putra *et al*. [31] studied the natural convection of nanofluids inside horizontal cylinder heated from one end and cooled from the other. An apparently paradoxical behavior of heat transfer deterioration was observed in the experimental study. The nature of this deterioration and its dependence on parameters such as particle concentration, material of the particles, and geometry of the containing cavity was investigated. The fluid characters are distinct from that of common slurries.

Heris *et al*. [32] dispersed CuO and Al_2_O_3 _oxide nanoparticles in water as base fluid in different concentrations, and the laminar flow convective heat transfer through circular tube with constant wall temperature boundary condition were examined. The experimental results obtained for CuO-water and Al_2_O_3_-water nanofluids indicate that heat transfer coefficient ratios for nanofluid to homogeneous model in low concentrations are close to each other, but by increasing the volume fraction, higher heat transfer enhancement for Al_2_O_3_/water was observed. The same authors worked on laminar flow forced convection heat transfer of Al_2_O_3_/water nanofluid inside a circular tube with constant wall temperature [33] and measured the Nusselt numbers for different nanoparticle concentrations as well as various Peclet and Reynolds numbers. Experimental results emphasized the enhancement of heat transfer due to the presence of nanoparticles in the fluid. Heat transfer coefficient increased by increasing the concentration of nanoparticles in nanofluid.

The turbulent convective heat transfer behavior of alumina (Al_2_O_3_) and zirconia (ZrO_2_) nanoparticle dispersions in water is investigated experimentally in a flow loop with a horizontal tube test section at various flow rates (9,000 < Re < 63,000) [34], The experimental data were compared to predictions made using the traditional single-phase convective heat transfer and viscous pressure loss correlations for fully developed turbulent flow, Dittus-Boelter, and Blasius/MacAdams, respectively. It was shown that if the measured temperature- and loading-dependent thermal conductivities and viscosities of the nanofluids are used in calculating the Reynolds, Prandtl, and Nusselt numbers, the existing correlations accurately reproduce the convective heat transfer and viscous pressure loss behavior in tubes. Therefore, no abnormal heat transfer enhancement was observed in this study.

Xuan and Li [35] conducted an experiment to investigate convective heat transfer and flow features of the nanofluid in a tube. Both the convective heat transfer coefficient and friction factor of the sample nanofluids for the turbulent flow were measured, respectively. The effects of such factors as the volume fraction of suspended nanoparticles and the Reynolds number on the heat transfer and flow features are discussed in detail. Wen and Ding [24] reported an experimental work on the convective heat transfer of nanofluids, made of γ-Al_2_O_3 _nanoparticles and DIW, flowing through a copper tube in the laminar flow regime. The results showed considerable enhancement of convective heat transfer using the nanofluids; the enhancement was particularly significant in the entrance region, and was much higher than that solely due to the enhancement on thermal conduction. The possible reasons for the enhancement are migration of nanoparticles and the resulting disturbance of the boundary layer.

You *et al*. [36] measured the critical heat flux (CHF) in the pool boiling of Al_2_O_3_-water nanofluids. They discovered an unprecedented phenomenon: a threefold increase in CHF over that of pure water. The average size of departing bubbles increased, and the bubble frequency decreased significantly in nanofluids when compared with those in pure water. Bang *et al*. [37] studied boiling heat transfer characteristics of Al_2_O_3_-based nanofluids. Pool boiling heat transfer coefficients and phenomena of nanofluids are compared with those of pure water, which are acquired on a smooth horizontal flat surface (roughness of a few tens of nanometers). The experimental results showed that these nanofluids have poor heat transfer performance compared to pure water in natural convection and nucleate boiling. On the other hand, CHF has been enhanced in not only horizontal but also vertical pool boiling. This is related to a change of surface characteristics by the deposition of nanoparticles.

Experimental study conducted by Das *et al*. [38] on pool boiling in water-Al_2_O_3 _nanofluids on horizontal tubes of small diameter revealed that the deterioration in performance in boiling is less in narrow tubes compared to that in large industrial tubes which makes it less susceptible to local overheating in convective application. Recently, Farajollahi *et al*. [39] measured heat transfer characteristics of γ-Al_2_O_3_/water and TiO_2_/water nanofluids in a shell and tube heat exchanger under turbulent flow condition. It was reported that by adding nanoparticles to the base fluid, significant enhancement of heat transfer characteristics was observed. For both nanofluids, two different optimum nanoparticle concentrations exist. A comparison of the heat transfer behavior of two nanofluids indicates that at a certain Peclet number, the heat transfer characteristics of TiO_2_/water nanofluid at its optimum nanoparticle concentration are greater than those of γ-Al_2_O_3_/water nanofluid while γ-Al_2_O_3_/water nanofluid possesses better heat transfer behavior at higher nanoparticle concentrations. In another recent study, it was demonstrated that the alumina nanofluids significantly improved the thermal performance of an oscillating heat pipe [40], with an optimal mass fraction of 0.9 wt.% for maximal heat transfer enhancement. Compared with pure water, the maximal thermal resistance was decreased by 0.14°C/W (or 32.5%) when the power input was 58.8 W at 70% filling ratio and 0.9% mass fraction. The authors observed that the nanoparticle settlement mainly took place at the evaporator. The change of surface condition at the evaporator due to nanoparticle settlement was found to be the major reason for the enhanced thermal performance of the alumina nanofluid-charged oscillating heat pipe.

Recently, Ho *et al*. [41], conducted an experiment on natural convection of heat transfer of a nanoluid in vertical square enclosures of different sizes, in the solid loading range of 0.1 to 4 vol.% noted the Rayleigh's number varying in the range of 6.21 × 10^3 ^to 2.56 × 10^8^. The experimental result for the average heat transfer rate across the three enclosures appeared generally consistent with the assessment based on the changes in thermophysical properties of the nanofluid formulated, showing systematic heat transfer degradation for the nanofluid containing nanoparticles volume fraction ≥2 vol.% over the entire range of the Rayleigh's number considered. The nanofluid containing 0.1 vol.%, a heat transfer enhancement of 18% compared with that of water, was found to arise in the largest enclosure at sufficiently high Rayleigh's number. The authors suggested that such enhancement is not only due to the relative changes in thermophysical properties of the nanofluid containing low particle fraction, other factors may come into play.

Although addition of local losses (orificing) may suppress instabilities, however, it is accompanied by a significant flow reduction which is detrimental to the natural circulation heat removal capability. Nayak *et al*. [42] demonstrated experimentally, with Al_2_O_3 _nanofluids, not only the flow instabilities are suppressed but also the natural circulation flow rate is enhanced. The increase in steady natural circulation flow rate due to addition of nanoparticles is found to be a function of its concentration in water. The flow instabilities are found to occur with water alone only during a sudden power addition from cold condition, step increase in power, and step decrease in power (step back conditions). With a small concentration of Al_2_O_3 _nanofluids, these instabilities are found to be suppressed significantly.

The heat transfer studies on alumina-based nanofluids can give rise to the possibility of their use in actual applications. However, cost of such fluids is a major concern *vis-à-vis *the stability duration of such fluids in ideal condition. Further, the effect of acids and bases or surfactants used for stabilization of nanoparticles in actual applications needs to be studied in detail.

### Applications of alumina-based nanofluids

A nanofluid can be used to cool automobile engines and welding equipment and to cool high heat flux devices such as high-power microwave tubes and high-power laser diode arrays. A nanofluid coolant could flow through tiny passages in MEMS too to improve its efficiency. The measurement of nanofluid CHF in a forced convection loop is useful for nuclear applications. If nanofluids improve chiller efficiency by 1%, a savings of 320 billion kWh of electricity or an equivalent 5.5 million barrels of oil per year would be realized in the USA alone [43]. Nanofluids find a potential for use in deep drilling application. A nanofluid can also be used for increasing the dielectric strength and life of the transformer oil by dispersing nanodiamond particles.

Nguyen *et al*. [44] experimentally investigated the behavior and heat transfer enhancement of an Al_2_O_3 _nanoparticle-water mixture, flowing inside a closed system that is destined for the cooling of microprocessors or other electronic components. Experimental data, obtained for turbulent flow regime, have clearly shown that the inclusion of nanoparticles into distilled water has produced a considerable enhancement of the cooling block convective heat transfer coefficient. For a particular nanofluid with 6.8% particle volume concentration, heat transfer coefficient has been found to increase as much as 40% compared to that of the base fluid. It has also been found that an increase of particle concentration has produced a clear decrease of the heated component temperature. Experimental data also showed that a nanofluid with a 36-nm particle provides higher heat transfer coefficients than a 47-nm particle size. In another experiment, You *et al*. [36] measured the enhancement of the CHF in pool boiling from a flat square heater immersed in alumina-based water nanofluid in a concentration range of 0 to 0.05 g/l. The test results showed that the enhancement of CHF was drastic when nanofluid was used as a cooling liquid instead of pure water. It was concluded that the increase in CHF levels present the possibility of raising chip power in electronic components or simplifying cooling requirements for space applications. Tzeng *et al*. [45] dispersed CuO and Al_2_O_3 _nanoparticles and antifoam, respectively into cooling engine oil for the cooling of automotive transmission. The experimental platform was a four-wheel drive transmission vehicle. It adopts advanced rotary blade coupling (RBC), where a high local temperature occurs easily at high rotating speeds. Therefore, it is imperative to improve the heat transfer efficiency. The experiment measures the temperature distribution of the RBC exterior at four different rotating speeds (400, 800, 1,200, and 1,600 rpm), simulating the conditions of a real car at different rotating speeds and investigating the optimum possible compositions of a nanofluid for higher heat transfer performance. Kulkarni *et al*. [46] used Al_2_O_3 _nanofluid as a coolant in a diesel electric generator. Specific heat measurements of aluminum oxide nanofluid with various particle concentrations were studied and showed that applying nanofluids resulted in a reduction of cogeneration efficiency. This is due to the decrease in specific heat, which influences the waste heat recovery from the engine. However, it was found that the efficiency of waste heat recovery heat exchanger increased for nanofluid due to its superior convective heat transfer coefficient.

Recently, Wu *et al*. [47] observed the potential of Al_2_O_3_-H_2_O nanofluids as a new phase change material for the thermal energy storage of cooling systems. The thermal response test shows the addition of Al_2_O_3 _nanoparticles remarkably decreases the super cooling degree of water, advances the beginning freezing time, and reduces the total freezing time. The infrared imaging photographs suggest that the freezing rate of nanofluids is enhanced and by only adding 0.2 wt.% Al_2_O_3 _nanoparticles, the total freezing time of Al_2_O_3_-H_2_O nanofluids can be reduced by 20.5%. Transformer cooling is important to the Navy as well as the power generation industry with the objective of reducing transformer size and weight. The ever growing demand for greater electricity production can lead to the necessity of replacing and/or upgrading transformers on a large scale and at a high cost. A potential alternative in many cases is the replacement of conventional transformer oil with a nanofluid. Such retrofits can represent considerable cost savings. It has been demonstrated that the heat transfer properties of transformer oils can be significantly improved by using nanoparticle additives [48].

The above experimental results demonstrated that alumina-based nanofluids have a significant potential in applications. However, large volumes of nanofluid experiments are lacking in literature. Further, most of the applications are limited to closed loop configuration. A need thus arises to test such fluids with suitable modifications in open loop applications.

### Summary

Alumina-based nanofluids are important because they can be used in numerous applications involving heat transfer and other applications. Most of the Al_2_O_3_-based nanofluids are prepared by using an ultrasonic vibrator which is not stable for a longer time.Researchers therefore had concentrated on preparing stable nanofluids by using different surfactants, optimizing pH, temperature for different nanofluids, and by surface modification of the particles. The thermal conductivity enhancement observed for Al_2_O_3 _nanofluid by different researchers is not consistent; the reason for this enhancement is not clear in the available literature. Very few literatures are available on the enhancement of thermal conductivity due to surface area, acidic or basic media, and due to the shape factor. The nanofluids prepared with acidic and basic media may not be useful for the heat transfer application, since it may cause adverse effects on the heat transfer properties. The effect of temperature observed by different authors demonstrates different degrees of enhancement for the same volume fraction. The technique for the measurement of thermal conductivity may also alter the values. The effect of temperature on thermal conductivity at lower volume fractions, which has been measured up to 400 K, has been reported . No work has yet been reported with experiments dealing with the measurement of thermal conductivity at low (sub-zero)-range temperatures. The behavior of the thermal conductivity at low temperatures are yet to be found out and can point a new direction in this field of research.

Very few reports described the effect of temperature on the viscosity at a higher volume fraction and at a higher temperature observing hysteresis phenomenon. The heating phase beyond the critical temperature may become more viscous which indicates a rather drastic alteration of the nanofluid rheological properties leading to hysteresis. Viscosity has raised a serious concern regarding the use of nanofluids for enhancing heat transfer mobility. Researchers can concentrate on the effect of temperature and the hysteresis behavior for Al_2_O_3 _nanofluids and can try to increase the temperature withstanding capacity of the Al_2_O_3 _nanofluids. From the observed results, it is clearly seen that nanofluids have a greater potential for heat transfer enhancement and are suitable for application in practical heat transfer processes.

The review has summarized the basics of nanofluid, its preparation methods, and the factors affecting the thermal conductivity enhancement in the Al_2_O_3_-based nanofluid. It has also identified the areas which require more research for better understanding. The enhancement of thermal conductivity of base fluid will be a definite requirement in the future to improve the thermal efficiency of different systems.

## Competing interests

The authors declare that they have no competing interests.

## Authors' contributions

SV compiled the studies conducted on thermal conductivity, viscosity, heat transfer and pool boiling phenomena of alumina based nanofluids, compared and analysed the results.

LNS involved in the conceptualizing the manuscript and revising it critically for improving through technical concepts. Both the authors read and approved the final manuscript.

## References

[B1] Lee S Choi SUS Li S Eastman JA Measuring thermal conductivity of fluids containing oxide nanoparticles ASME J Heat Transfer 1999 121 280 89 10.1115/1.2825978

[B2] Wang XQ Mujumdar AS A review on nanofluids - part I: theoretical and numerical investigations Braz J Chem Eng 2008 25 613 630

[B3] Singh AK Thermal conductivity of nanofluids Defence Sci J 2008 58 600 607

[B4] Sridhara V Gowrishankar BS Snehalatha C Satapathy LN Nanofluids--a new promising fluid for cooling Trans Ind Ceram Soc 2009 68 1 17

[B5] Paul G Chopkar M Manna IA Das PK Techniques for measuring the thermal conductivity of nanofluids: a review Renew Sust Energ Rev 2010 14 1913 1924 10.1016/j.rser.2010.03.017

[B6] Godson L Raja B Mohan Lal D Wongwises S Enhancement of heat transfer using nanofluids--an overview Renew Sust Energ Rev 2010 14 629 641 10.1016/j.rser.2009.10.004

[B7] Das SK Putra N Thiesen P Roetzel W Temperature dependence of thermal conductivity enhancement for nanofluids ASME J Heat Transfer 2003 125 567 574 10.1115/1.1571080

[B8] Wang X Xu X Choi SUS Thermal conductivity of nanoparticle-fluid mixture J Thermophys Heat Trans 1999 13 474 480 10.2514/2.6486

[B9] Xuan Y Li Q Heat transfer enhancement of nanofluids Int J Heat Fluid Fl 2000 21 58 64 10.1016/S0142-727X(99)00067-3

[B10] Murshed SMS Leong KC Yang C Enhanced thermal conductivity of TiO_2_--water based nanofluids Int J Therm Sci 2005 44 367 373 10.1016/j.ijthermalsci.2004.12.005

[B11] Beck MP Sun T Teja AS The thermal conductivity of alumina nanoparticles dispersed in ethylene glycol Fluid Phase Equilib 2007 260 275 278 10.1016/j.fluid.2007.07.034

[B12] Choi C Yoo HS Oh JM Preparation and heat transfer properties of nanoparticles-in-transformer oil dispersions as advanced energy-efficient coolants Curr Appl Phys 2008 8 710 712 10.1016/j.cap.2007.04.060

[B13] Li CH Peterson GP Experimental investigation of temperature and volume fraction variations on the effective thermal conductivity nanoparticle suspensions (nanofluids) J Appl Phys 2006 99 084314 10.1063/1.2191571

[B14] Timofeeva EV Gavrilov AN McCloskey JM Tolmachev YV Thermal conductivity and particle agglomeration in alumina nanofluids: experiment and theory Phys Rev E 2007 76 061203 10.1103/PhysRevE.76.061203 18233838

[B15] Masuda H Ebata A Teramae K Hishinuma N Alteration of thermal conductivity and viscosity of liquid by dispersing ultra-fine particles (dispersion of γ-Al_2_O_3_, SiO_2_, and TiO_2 _ultra-fine particles) Netsu Bussei 1993 7 227 233

[B16] Xie H Wang J Xi T Liu Y Ai F Thermal conductivity enhancement of suspensions containing nanosized alumna particles J Appl Phys 2002 91 4568 4572 10.1063/1.1454184

[B17] Hwang D Hong KS Yang HS Study of thermal conductivity nanofluids for the application of heat transfer fluids Thermochim Acta 2007 455 66 69 10.1016/j.tca.2006.12.006

[B18] Eastman JA Choi SUS Li S Yu W Thomson LJ Anomalously increased effective thermal conductivities of ethylene glycol-based nanofluids containing copper nanoparticles Appl Phys Lett 2001 78 718 720 10.1063/1.1341218

[B19] Lee JH Hwang KS Jang SP Lee BH Kim JH Choi SUS Choi CJ Effective viscosities and thermal conductivities of aqueous nanofluids containing low volume concentrations of Al_2_O_3 _nanoparticles Int J Heat Mass Trans 2008 51 2651 656 10.1016/j.ijheatmasstransfer.2007.10.026

[B20] Murshed SMS Leong KC Yang C Invesitions of thermal conductivity and viscosity of nanofluids Int J Therm Sci 2008 47 560 568 10.1016/j.ijthermalsci.2007.05.004

[B21] Yu W France DM Routbort JL Choi SUS Review and comparison of nanofluid thermal conductivity and heat transfer enhancements Heat Transfer Eng 2008 29 432 460 10.1080/01457630701850851

[B22] Beck MP Yuan Y Warrier P Teja AS The thermal conductivity of alumina nanofluids in water, ethylene glycol, and ethylene glycol + water mixtures J Nanopart Res 2010 12 1469 1477 10.1007/s11051-009-9716-9

[B23] Oh DW Jain A Eaton JK Goodson KE Lee JS Thermal conductivity measurement and sedimentation detection of aluminum oxide nanofluids by using 3ω method Int J Heat Fluid Fl 2008 29 1456 1461 10.1016/j.ijheatfluidflow.2008.04.007

[B24] Wen D Ding Y Experimental investigation into convective heat transfer of nanofluids at the entrance region under laminar flow conditions Int J Heat Mass Trans 2004 47 5181 5188 10.1016/j.ijheatmasstransfer.2004.07.012

[B25] Kole M Dey TK Thermal conductivity and viscosity of Al_2_O_3 _nanofluid based on car engine coolant J Phys D Appl Phys 2010 43 315501 10.1088/0022-3727/43/31/315501

[B26] Das SK Putra N Roetzel W Pool boiling characteristics of nano-fluids Int J Heat Mass Trans 2003 46 851 862 10.1016/S0017-9310(02)00348-4

[B27] Liu MS Lin MC Huang IT Wang CC Enhancement of thermal conductivity with CuO for nanofluids Chem Eng Technol 2006 29 72 77 10.1002/ceat.200500184

[B28] Nguyen CT Desgranges F Roy G Galanis N Mare T Boucher S Angue Mintsa H Viscosity data for Al_2_O_3_-water nanofluid--hysteresis: is heat transfer enhancement using nanofluids reliable? Int J Heat Fluid Fl 2007 28 1492 506 10.1016/j.ijheatfluidflow.2007.02.004

[B29] Xie H Chen L Wu Q Measurements of the viscosity of suspensions (nanofluids) containing nanosized Al_2_O_3 _particles High Temp-High Press 2008 37 127 135

[B30] Pak BC Cho YI Hydrodynamic and heat transfer study of dispersed fluids with submicron metallic oxide particles Exp Heat Tran 1998 11 151 170 10.1080/08916159808946559

[B31] Putra N Roetzel W Das SK Natural convection of nanofluids Heat Mass Transf 2003 39 775 784 10.1007/s00231-002-0382-z

[B32] Heris SZ Etemad SG Esfahan MN Experimental investigation of oxide nanofluids laminar flow convective heat transfer Int Commun Heat Mass Trans 2006 33 529 535 10.1016/j.icheatmasstransfer.2006.01.005

[B33] Heris SZ Esfahany MN Etemad SG Experimental investigation of convective heat transfer of Al_2_O_3_/water nanofluid in circular tube Int J Heat Fluid Fl 2007 28 203 210 10.1016/j.ijheatfluidflow.2006.05.001

[B34] Williams W Buongiorno J Wen Hu L Experimental investigation of turbulent convective heat transfer and pressure loss of alumina/water and zirconia/water nanoparticle colloids (Nanofluids) in horizontal tube J Heat Trans 2008 130 042412 10.1115/1.2818775

[B35] Xuan Y Li Q Investigation on convective heat transfer and flow features of nanofluid J Heat Trans 2003 125 151 155 10.1115/1.1532008

[B36] You SM Kim JH Kim KM Effect of nanoparticles on critical heat flux of water in pool boiling heat transfer Appl Phys Lett 2003 83 3374 3376 10.1063/1.1619206

[B37] Bang IC Chang SH Boiling heat transfer performance and phenomena of Al_2_O_3_-water nano-fluids from a plain surface in a pool Int J Heat Mass Trans 2005 48 2407 2419 10.1016/j.ijheatmasstransfer.2004.12.047

[B38] Das SK Putra N Roetzel W Pool boiling of nano-fluids on horizontal narrow tubes Int J Multiphase Fl 2003 29 1237 1247 10.1016/S0301-9322(03)00105-8

[B39] Farajollahi B Etemad SG Hojjat M Heat transfer of nanofluids in a shell and tube heat exchanger Int J Heat Mass Trans 2010 53 12 17 10.1016/j.ijheatmasstransfer.2009.10.019

[B40] Qu J Wu HY Cheng P Thermal performance of an oscillating heat pipe with Al_2_O_3_-water nanofluids Int Commun Heat Mass Trans 2010 37 111 115 10.1016/j.icheatmasstransfer.2009.10.001

[B41] Ho CJ Liu WK Chang YS Lin CC Natural convection heat transfer of alumina-water nanofluid in vertical square enclosures: an experimental study Int J Therm Sci 2010 49 1345 1353 10.1016/j.ijthermalsci.2010.02.013

[B42] Nayak AK Gartia MR Vijayan PK Thermal-hydraulic characteristics of a single-phase natural circulation loop with water and Al_2_O_3 _nanofluids Nucl Eng Des 2009 239 526 540 10.1016/j.nucengdes.2008.11.014

[B43] Nanofluids http://www.uitonline.it/doc/NANO-FLUIDS.pdf

[B44] Nguyen CT Roy G Gauthier C Galanis N Heat transfer enhancement using Al_2_O_3_-water nanofluid for an electronic liquid cooling system Appl Therm Eng 2007 27 1501 1506 10.1016/j.applthermaleng.2006.09.028

[B45] Tseng SC Lin CW Huang K Heat transfer enhancement of nanofluids in rotary blade coupling of four wheel-drive vehicles Acta Mechanica 2005 179 11 23 10.1007/s00707-005-0248-9

[B46] Kulkarni DP Vajjha RS Das DK Oliva D Application of aluminum oxide nanofluids in diesel electric generator as jacket water coolant Appl Therm Eng 2008 28 1774 1781 10.1016/j.applthermaleng.2007.11.017

[B47] Wu S Zhu D Li X Li H Le J Thermal energy storage behavior of Al_2_O_3_-H_2_O nanofluids Thermochim Acta 2009 483 73 77 10.1016/j.tca.2008.11.006

[B48] Wang XQ Mujumdar AS A review on nanofluids - part II: experiments and applications Braz J Chem Eng 2008 25 631 648 10.1590/S0104-66322008000400002

